# Altered Expression of RB and pRB in Tissue Arrays of Primary Breast Cancers and Matched Axillary Lymph Node Metastases

**DOI:** 10.1155/2022/5221257

**Published:** 2022-03-04

**Authors:** Carmen Leser, Angelika Reiner, Georg Dorffner, Marie-Theres Kastner, Martin Igaz, Christian Singer, Deirdre Maria König-Castillo, Christine Deutschmann, Daniel König, Iris Holzer, Daphne Gschwantler-Kaulich

**Affiliations:** ^1^Department of Obstetrics and Gynecology, Cancer Comprehensive Center, Medical University of Vienna, Vienna, Austria; ^2^Department of Pathology, Danube Hospital, Vienna, Austria; ^3^Section for Artificial Intelligence and Decision Support, Medical University of Vienna, Vienna, Austria; ^4^Laboratory Dr. Kosak, Vienna, Austria; ^5^Department of Urology, Medical University of Vienna, Vienna, Austria; ^6^Clinical Division of Social Psychiatry, Department of Psychiatry and Psychotherapy, Medical University of Vienna, Vienna, Austria

## Abstract

**Objectives:**

The retinoblastoma (RB) pathway is crucial in the development and progression of many cancers. To better understand the biology of progressive breast cancer (BC), we examined protein expression of the RB pathway in primary BCs and matched axillary lymph node metastases (LM).

**Methods:**

Immunohistochemistry was used to evaluate cyclin D1, CDK4/6, RB, phosphorylated RB (pRB), and E2F1 expression in tissue arrays containing cores of 50 primary BCs and matched LM. The number of positive tumor cells and staining intensity were scored.

**Results:**

The proteins were localized in the nucleus, while CDK6 was detected in the cytoplasm and CDK4 was found in both. pRB and E2F1 showed higher expression in matched LM than in primary tumors. Expression of these proteins differed significantly by the percentage of positive tumor cells, while proteins in the proximal portion of the RB pathway showed no significant differences. The main path of alteration consisted of high pRB in primary BC, remaining pRB high in the majority of LM, variations occurring in fewer cases. All matched LM of the few primary tumors that had unaltered RB and pRB expression showed changes in RB or pRB expression.

**Conclusion:**

Expression of pRB and E2F1 was significantly higher in LM than in primary BC. A majority of cancers with LM showed altered RB or pRB expression, suggesting that proteins downstream in the RB pathway play a critical role in metastatic BC and disease progression. So looking at the RB pathway could be an option for chemotherapy decisions in patients with only few LM.

## 1. Introduction

**Figure 1:**
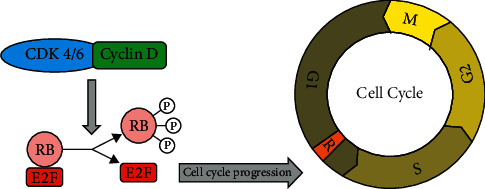
Cyclin D cyclin-dependent kinase 4/6 retinoblastoma pathway.

Accounting for 25% of malignant neoplasms in women worldwide, breast cancer (BC) is the most common malignant tumor in women [1]. The retinoblastoma (RB) pathway (Figure 1) plays a critical role in all types of BCs; however, the mechanisms by which the pathway is inactivated vary. Luminal BC is the most common subtype of BC and is treated with endocrine therapy or surgical resection. In most cases, luminal BC has a favorable prognosis with no recurrence, but approximately 30% of these patients suffer from it. It may be due to the development of endocrine resistance and evasion of endogenous growth suppressors [2]. The mechanism underlying such a recurrence is not fully understood, but alterations in tumor pathways and cell cycle checkpoints are known to contribute [3]. Dysregulation of CDK4/6 is a common phenomenon in luminal BC recurrence and is a result of alterations in the RB pathway caused by increased expression of cyclin D1 [4]. Based on this, therapeutics targeting the cyclin D-CDK4/6-Rb pathway have gained interest as treatments for luminal BC [5]. Her2 positive BC is often associated with cyclin D1 deregulation, but rarely with RB loss [6]. Contrarily, dysregulation of the RB pathway is caused mainly through RB loss in triple-negative BC (TNBC), and RB loss in TNBC is associated with good responses to chemotherapy [7].

Despite these interesting findings, neither clinical use nor standardized methodologies for analysis of RB pathway deregulation have been established. Surprisingly, there have been few analyses of the RB pathway in clinical specimens. While some data showing RB pathway deregulation in early BC such as in ductal carcinoma *in situ*, does exist [8], RB pathway disruption in metastasized BC has not been described to the best of our knowledge. Therefore, we investigated the expression of proteins in the proximal and downstream portions of the RB pathway in lymphnode positive tumors, comparing primary BC and LM.

## 2. Methods

### 2.1. Patient and Tumor Characteristics

A total of 47 invasive ductal carcinomas and three invasive lobular carcinomas were examined. The clinicopathological parameters of the tumors were provided by the distributor and are detailed in Table 1. Altered expression of RB and pRB was assumed as defined by Gravessea et al. [9].

### 2.2. Immunohistochemistry

All markers were investigated by immunohistochemistry (IHC) on formalin fixed paraffin embedded slides containing 50 tissue invasive BCs and 50 matched LM cores using commercially available tissue microarrays (BR 10010e, US Biomax Inc., Rockville, MD, USA). Tissue microarrays consisted of duplicate cores per case with 1 mm in diameter each.

Tumor characterization for steroid hormone receptors and immunohistochemical scoring for HER-2 were used as given by the provider. Data for gene amplification was not given. Thus, cases with equivocal HER-2 scoring were excluded for some correlations in results.

Discrepancies of case numbers are due to missing values because some tissue cores came off the slides during laboratory processing.

All IHC analyses were performed on a Ventana® BenchMark System using the OptiView DAB IHC Detection Kit (Ventana Medical Systems, Inc., Oro Valley, Arizona, USA). Incubation omitting primary antibodies was used as a negative control. Descriptions of primary antibodies are provided in Table 2. Antigen retrieval before application of the first antibody was carried out by heat incubation (37°C). As indicated in Table 2, some of the antibodies required additional incubation per the instructions of the Ventana OptiView Amplification Kit.

Microscopic analysis was performed by scoring the number of positive tumor cells semiquantitatively for all markers. The percentage of positive tumor cells was normalized to the total number of tumor cells. Stromal cells like fibroblasts or nontumorigenic tissue were not taken into account. Samples were divided into three groups based on the number of stained tumor cells: negative (0% of tumor cells stained), low (≤50% of tumor cells stained), and high (>50% of tumor cells stained). Nuclear staining was scored for cyclin D1, CDK4, RB, pRB, and E2F1. CDK6 showed only cytoplasmic staining. Any positive staining was considered a positive result. In a few cases with discrepancies of immunohistochemical staining, higher protein expression in either core was considered for the result.

### 2.3. Statistical Analysis

This study was strictly exploratory in nature; thus, no correction for multiple testing was applied. A significance level of *α* = 0.05 was used for reporting and interpretation of clinical plausibility and consistency of results.

Statistical analyses were performed to determine the statistical significance of observed differences in staining between the primary tumor and corresponding lymph node metastases for each antibody. Where significant differences were observed, intergroup differences in BC subgroups (i.e., TNBC, ER, PR, or HER-2 positive tumors) were investigated.

Either the chi-squared test or Fisher's exact tests were used to assess intergroup differences in expression. The associations between the expression of tested markers and clinicopathological parameters were analyzed by Spearman's Rho test. All calculations were performed using IBM SPSS Statistics version 23 (IBM Corp., Armonk, NY).

## 3. Results

**Figure 2:**
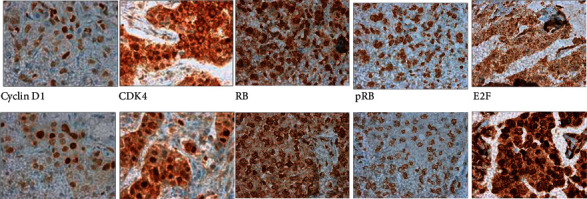
Immunohistochemical staining of primary breast cancer and matched axillary lymph node metastases. Original magnification x400. First row shows primary tumors, and second row shows matched axillary lymph node metastases.

All proteins were present in the nucleus (Figure 2). Expression of cyclin D1 and CDK4 was significantly correlated (correlation coefficient 0.4).

**Figure 3:**
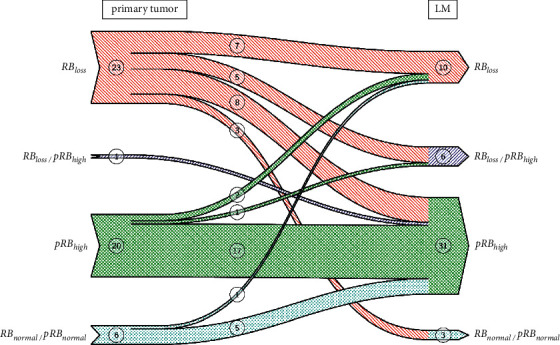
Change of RB and pRB from primary tumor to lymph node metastasis.

Positive expression of proteins in the proximal RB pathway (cyclin D1, CDK4, and RB) was comparable. However, proteins in the downstream portion of the RB pathway (pRB, E2F1) showed significantly higher positivity in LM than in BC (Table 3). In the majority of primary BCs and LM, pRB was expressed at high levels, but above 50%. The majority of cases in E2F1 showed expression at low levels, below 50%. Altered expression of RB and pRB was observed in 43 primary BC, reflecting a disrupted RB pathway. The alteration to the RB pathway was due to RB loss in 23 primary BCs (negative immunohistochemical staining) and high levels of pRB expression in 21 primary BCs (Table 4). Only one of the cancers with altered expression had both RB loss and high positivity for pRB. The change of RB and pRB from primary tumor to lymph node metastasis is depicted in Figure 3. In primary BC with high levels of pRB expression, pRB expression was also high in matched axillary lymph node metastases. In contrast, primary BC with RB loss showed a variety of changes in LM at comparable frequencies. All primary BC with unaltered RB or pRB showed altered RB or pRB in all matched LM. In subgroup analyses, cancers of all molecular subtypes exhibited altered RB (15 of 19 luminal, 5 of 5 HER-2 positive, 13 of 16 TNBC, and all 9 Her-2 equivocal cases). Only eight of the cancers showed unaltered expression of RB or pRB in primary BC, most likely suggesting an intact RB pathway. Among these, four were ER negative and four were ER positive. Among the matching LM, seven showed high expression of pRB and two showed RB loss. This suggests RB pathway disruption in lymph node metastases of primary BC cases with unaltered RB pathway protein expression.

Correlations between protein expression and tumor grade are provided in Table 5. Only proteins in the downstream portion of the RB pathway (pRB and E2F1) showed different expressions in different tumors. RB and pRB were not expressed in G1 cancers and were equally expressed in G2 in the majority of G3 cancers presenting positive expression (Table 5).

Proteins in the downstream portion of the RB pathway (RB and pRB) were also evaluated based on the level of lymph node metastases (1–3 LM; N1; 4–9 LM, N2). The vast majority showed altered RB with no differences between N1 and N2 (Table 6).

An overwhelming majority of luminal cancers showed positivity for all proteins; this was also true for HER-2 negative cancers. In TNBCs, all proteins showed roughly comparable distributions (Table 7). For HER-2 Score 3+ and thus, most likely, HER-2 amplified BC, the number of cases was too small for conclusive interpretation.

## 4. Discussion

The RB protein functions as a tumor suppressor, and deregulation of the RB pathway plays a critical role in tumor development and disease progression. In its nonphosphorylated active form, RB binds to the transcription factor E2F1 to repress progression from the G1 to the S phase. Thus, RB is an important gatekeeper in cell growth [10]. Cyclin D1 binds and regulates CDK4, which deactivates numerous targets including RB. E2F1 dissociation from the RB/E2F complex leads to loss of the growth-inhibiting function of RB (Figure 2) [3].

Interestingly, RB pathway disruption is an important part of the progression of intraductal BC and is therefore relevant to the early stages of invasive BC [8]. This suggests that changes in the RB pathway may be associated with malignant transformation, even in the early stages of BC development, and also play a significant role in tumor progression in later stages of disease. Changes in the RB pathway may also serve as therapeutic indicators, but no predictive test using RB pathway alteration has been established. Development of a single specific molecule test may be difficult due to the variability in the effects of RB pathway alterations associated with different BC subtypes. Despite this, monitoring RB pathway deregulation could be clinically relevant for the development of individualized treatments in the future. To contribute to a deeper understanding of RB pathway disruption, we have analyzed the expression of proteins in the RB pathway in primary BC and matched axillary lymph node metastases.

Our results showed a significantly higher number of samples that showed expression of pRB and E2F1 in greater than 50% of the samples in LM compared with primary BC tumors. This altered protein expression occurs downstream in the pathway and reflects enhanced cell cycle progression. In contrast, expression of proteins in the proximal pathway was not significantly different in the primary tumors and metastases. We also examined protein expression patterns reflective of an altered or disrupted pathway as described by Gavressea et al. [9]. In our study, the overwhelming majority of primary cancers had altered protein expression leading to either RB loss or high levels of pRB. Few primary cancers showed no alterations in protein expression, but all BCs that had no alterations in RB pathway protein expression exhibited altered RB or pRB in their matched LM lymph node metastases. In 2017, Gavressa et al. found that the p53 mediated sensitivity of breast cancer cells to chemotherapeutic agents appears to be driven mostly by pRB, and that using agents that enhance RB phosphorylation might possibly increase the chemosensitivity of breast cancer cells. This may corroborate the wide acceptance of chemotherapy as an adjuvant therapy regimen, especially chemotherapy in BC with axillary lymph node metastases. Our findings suggest that chemotherapy should be an option for patients with few lymph node metastases. Derenzini et al. have previously shown that human BC cells exhibiting RB loss have higher sensitivity to 5-FU and methotrexate [11]. However, some studies have found that low expression of RB in ER+/HER2+ patients leads to low pCR rates after neoadjuvant chemotherapy with anti-HER2 drugs [12, 13]. Furthermore, in ER+/HER2 negative BC, RB loss of function is a predictive biomarker of resistance to palbociclib [14].

No significant correlations were observed between protein expression and tumor grade, but the majority of G3 primary tumors exhibited altered expression of pRB and E2F1, indicating enhanced cell cycle progression. Interestingly, this finding does not correlate with other literature showing that RB expression correlates with histologic grades 1 and 2 [15]. This difference can likely be explained by the fact that this earlier study examined only TNBCs, which are the most poorly differentiated subtype of BCs, whereas the current study included all molecular subtypes and tumor grades. Unlike earlier studies [16, 17], we did not observe any significant correlations between molecular tumor subtypes. The lack of correlations in our study may be due to the small number of samples of each subtype included. In the commercially available TMA, HER-2 score 2+ cases were overrepresented compared to unselected BC cases. No data exist for these cases following HER-2 in situ hybridization results, according to the clinical guidelines. It should also be noted that in cases included in the commercially available TMAs, only small tumor samples are examined, which may result in negative staining in cases of pronounced protein expression heterogeneity. These shortcomings in our study may have contributed to the discrepancies between our results and earlier studies, mainly consisted of HER-2 of score 2+ with unknown in situ hybridization results.

The results of our study are limited because of the small sample size and partly limited patient characteristics given by the provider of tissue microarrays. However, due to our promising findings, we plan a future study on the tissue of patients from our institution. In former studies, some markers have been shown to be quite heterogeneous in breast carcinomas. Some discrepancies between primary tumors and metastases could be due to tumor heterogeneity [18].

In conclusion, increased expression of E2F and pRB in lymph node metastasis compared with primary BC indicates that the downstream part of the cyclin D-CDK4/6-RB pathway is more often activated in metastatic tissue. In addition, a majority of cancers with axillary lymph node metastasis showed alterations of RB and pRB. This suggests proteins downstream of the RB pathway play a critical role in metastatic breast cancer and progressive disease. Therefore, evaluation of the RB pathway may be prognostically relevant.

## Figures and Tables

**Table 1:** Clinicopathologic parameters.

Clinicopathological characteristics	*n* (%)
Median age in years (IQR)	50 (44–55)
*Histological subtype*	
Invasive ductal carcinoma	47 (94%)
Invasive lobular carcinoma	3 (6%)
*Grade*	
X	6 (12%)
1	7 (14%)
2	28 (54%)
3	9 (18%)
*Tumor stage*	
T1	1 (2%)
T2	32 (64%)
T3	10 (20%)
T4	7 (14%)
*Nodal status*	
N1	29 (58%)
N2	20 (40%)
N3	1 (2%)
*Receptors*	
Triple negative	16 (32%)
Hormone receptor positive (mainly ER, partly PR)	19 (38%)
HER-2 equivocal (score 2+)	9 (18%)
HER-2 positive (score 3+)	5 (10%)
Unknown	1 (2%)

**Table 2:** Primary antibodies used in immunohistochemistry.

Antibody	Clone	Dilution	Company	Pretreatment
Cyclin D1	SP4-R	Ready to use	Ventana® Medical Systems	CC1 Ventana® Medical Systems
CDK4	DCS-156	1 : 5	©Zytomed Systems	CC1 and OptiView Amplification Kit
RB	1F8 (Rb1)	1 : 50	Thermo Fisher Scientific	CC1 and OptiView Amplification Kit
pRB	pThr826	1 : 100	Thermo Fisher Scientific	CC1 and OptiView Amplification Kit
E2F1	KH95	1 : 80	Thermo Fisher Scientific	CC1 and OptiView Amplification Kit

**Table 3:** RB pathway proteins in primary BC compared with LM.

	Primary cancer		LM		*χ*2
	Positive	Negative	Positive	Negative	
Cyclin D1	43 (86%)	7 (14%)	38 (83%)	8 (17%)	Ns
CDK4	28 (56%)	22 (44%)	21 (42%)	29 (58%)	Ns
RB	27 (54%)	23 (46%)	32 (64%)	18 (36%)	Ns
pRB	27 (54%)	23 (46%)	43 (86%)	7 (14%)	0.0004
E2F1	33 (69%)	15 (31%)	43 (86%)	7 (14%)	0.041

**Table 4:** Retinoblastoma pathway proteins with significantly different expression in primary breast cancer and matched axillary lymph node metastases.

	Primary cancer			LM		
	0	<50%	>50%	0	<50%	>50%
pRB	23 (46%)	7 (14%)	20 (40%)	7 (14%)	6 (12%)	37 (74%)
E2F1	15 (31%)	26 (54%)	7 (15%)	7 (14%)	35 (70%)	8 (16%)

*Note.* Different numbers are due to missing cores after staining.

**Table 5:** RB pathway biomarkers in different grades of primary breast cancer tumors.

Primary BC	Grade 1		Grade 2		Grade 3	
	Positive	Negative	Positive	Negative	Positive	Negative
Cyclin D1	6	1	25	3	7	2
CDK4	3	4	16	12	5	4
RB	1	6	16	12	6	3
pRB	0	7	16	12	8	1
E2F1	4	3	19	8	7	2

**Table 6:** Protein expression of the downstream RB pathway in axillary lymph node metastasis.

	N1	N2
	*n* = 28	*n* = 20
RB loss	15	9
pRB high positivity	12	10
Overlap	1	1

**Table 7:** RB pathway biomarkers in BC subtypes.

	ER		TNBC		HER-2			
					0/1+		3+	
	Positive	Negative	Positive	Negative	Positive	Negative	Positive	Negative
Cyclin D1	18	1	12	4	26	4	5	0
CDK4	14	5	6	10	16	14	1	4
RB	12	7	9	7	17	13	2	3
pRB	12	7	9	7	17	13	2	3
E2F1	15	3	8	7	19	10	3	2

*Note.* Different numbers are due to missing values.

## Data Availability

All the necessary data are included within the article.
